# Syria: What should health care professionals do?

**DOI:** 10.7189/jogh.04.010302

**Published:** 2014-06

**Authors:** Amelia Martin, Nathan Post, Martha Martin

**Affiliations:** 1 School of Medicine, University of Bristol, Bristol, United Kingdom; 2 Medsin-UK, student-led registered charity focused on global health equity; 3 School of Medicine, Newcastle University, Newcastle, United Kingdom; 4 School of Medicine, King's College London, London, United Kingdom

The Syrian conflict started in March 2011 with civil unrest and has now progressed to civil war [1]. The death toll stands at more than 100 000 men, women and children [2]. The UN Human Rights Council has accused both the Syrian government and the opposition of committing war crimes and crimes against humanity [3]. Within a few years, Syria has evolved from the world’s second largest refugee-hosting country, to fast becoming the largest refugee-producing country [4]. UNHCR has estimated there are 9.3 million people, 1 million of these children, in need. 6.3 million Syrians are internally displaced – a number that is expected to rise if the conflict continues unabated [4]. In these unstable conditions, health is one of the biggest concerns for these people [5].

With the conflict continuing unabated, inability to access health care is now a reality of daily life for millions of people. Syria was widely considered to have a well functioning health system before the conflict started [6]. However, with many millions of displaced, vulnerable people requiring all levels of health care, the fragile health system is under threat. This is not simply an issue of burden, but of two fundamental, catastrophic problems that must be addressed by the international community: a blocking of sufficient humanitarian aid and deliberate, direct attacks on the health care system, which are being widely used as a weapon of war [7].

Health is a right for all [8]. For this reason, hospitals, medical units and health care personnel require special protection in times of crisis. Additionally, to enable the capacity of the health system to reach adequate levels, channels of humanitarian assistance must be opened, including cross-border aid and removing control solely from the hands of the Government [9].

In Syria, the deliberate targeting of these providers is a distinct and chilling reality [10]. Numerous reports have highlighted the forcible denial of care to sick and wounded Syrian civilians [10]. According to the UN, almost two thirds of public hospitals are unable to function, and as many emergency ambulances have been rendered unable to provide services to the public. [11]. Of grave concern are reports of the deliberate targeting of health workers: in the context of wider abuses of human rights and crimes against humanity, health personnel face detention if found to be providing treatment or even simply carrying medicines [10,12].

Such destruction of the infrastructure, resources and workforce of the health system surely presents an exacerbated challenge for post-conflict rehabilitation. A growing body of evidence points to the importance of a strong health system in successful reconstruction of other sectors and the country as a whole [13].

As medical students, one development of the systematic targeting of health care is of particular concern. Two fourth year medical students from Aleppo University were recently arrested whilst working with a team in a field hospital treating injured demonstrators. According to Amnesty International, their bodies were later found burned and mutilated: one of the students had his hands bound and had suffered a gunshot wound to the head [14].

This threat to the depoliticised nature of humanitarian relief, or more significantly a threat to the provision of essential health care, should be something the health professions fight to defend against. As medical students, we have a unique mandate and responsibility to speak out against such atrocities against our colleagues in Syria, and call the UN to action over the position it has already adopted in promoting International Humanitarian Law, human rights and supporting the Declaration of Geneva [10].

**Figure:**
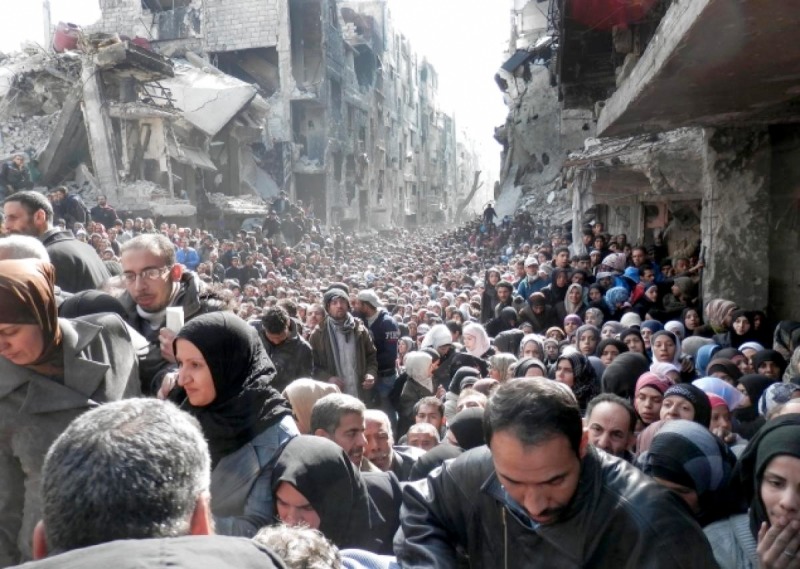
Photo: UN photo from UNRWA archives, January 2014

In March, the International Federation of Medical Students Associations, the IFMSA, passed two policies relevant to the Syrian conflict: the first calls for action from international actors to do more to protect health services and the health workforce in conflict situations [15]. The second calls for international actors to fulfill their funding commitments for the Syrian crisis, and supports the MSF open letter calling for more open channels of humanitarian assistance and medical aid [16]. This complements the UN Security Council Resolution 2139 in 2014 to increase humanitarian access and aid delivery in Syria. In addition to respecting the principle of medical neutrality to comply with international humanitarian law, that the wounded and sick must receive medical care and attention required by their condition [17]. These policies, advocating on behalf of over 1.4 million medical students from 114 countries, represent a significant call to action for international actors to do more to preserve the health of Syrians.

Through the IFMSA, international collaboration has been initiated, which sets a precedent for other professional networks and international actors to do the same. UK medical students will work with their colleagues from Jordan, Lebanon, Kuwait and Iraq to support Syrian civilians in need. This represents the ability for health professionals from all over the world to present a united voice speaking out against human rights violations and a threat to health care. We call for the UN to turn statements into action.

## References

[1] Margesson R, Chesser S. Syria: Overview of the Humanitarian Response. Congressional Research Service. 2014; 7-5700:1-24.

[2] UN OCHA Syria Crisis. Available at: http://syria.unocha.org Accessed: 30 January 2014.

[3] Amnesty International Annual Report, Syria 2013. Available at: http://www.amnesty.org/en/region/syria/report-2013 Accessed: 30 January 2014.

[4] Guterres A. UN Refugee Agency. Donor nations pledge US$2.4 billion at Kuwait meet for Syrians in need. Available at:http://www.unhcr.org/52d6ca0f6.html Accessed: 30 January 2014.

[5] Lancet (2013). The right to health for Syrian refugees.. Lancet.

[6] Medecins Sans Frontieres (2013). From birth to death in Syria.. Alert MSF Publication..

[7] Medecins sans Frontieres. Syria: Urgent need for cross border aid for Syrians. Available at: http://www.doctorswithoutborders.org/press/release.cfm?id=7229&cat=press-release#sthash.gERZ0Grv.dpuf Accessed: 30 January 2014.

[8] World Health Organisation. The Right to Health, Fact sheet N°323Reviewed November 2013, Available at: http://www.who.int/mediacentre/factsheets/fs323/en/ Accessed 25 March 2014.

[9] Medecins Sans Frontieres. An Open letter to the Member States of the High Level Group on Syria. Available at: http://www.doctorswithoutborders.org/publications/article.cfm?id=7228&cat=open-letters#sthash.t5mdzWef.dpuf Accessed: 31 January 2014.

[10] Human Rights Council. Human rights situations that require The Council’s attention, assault on medical care in Syria. Available at: http://www.ohchr.org/EN/HRBodies/HRC/RegularSessions/Session24/Documents/A-HRC-24-CRP-2.doc Accessed: 15 February 2014.

[11] UN News Centre. Syria: Senior UN officials strongly condemn attacks on health personnel, facilities, damaged. Available at: http://www.un.org/apps/news/story.asp?NewsID=46683&Cr=Syria&Cr1=#.UyzJrrIgGSP Accessed: 15 February 2014.

[12] Medecins Sans Frontieres. Syria: When medicine is made a weapon. Available at: http://www.doctorswithoutborders.org/article/syria-when-medicine-made-weapon Accessed: 16 February 2014.

[13] Kruk ME (2010). Rebuilding health systems to improve health and promote statebuilding in post-conﬂict countries: A theoretical framework and research agenda.. Soc Sci Med.

[14] Amnesty International. Syria: Detained medics tortured and killed amid Aleppo crackdown. Available at: http://www.amnesty.org/en/news/syria-detained-medics-killed-brutal-bid-silence-dissent-2012-06-26 Accessed: 20 February 2014.

[15] International Federation of Medical Students Association Standing Committee on Humans Rights and Peace. Protection of Health Services Policy Statement. International Federation of Medical Students Association Conference March meeting. Hammamet, Tunisia. 2014

[16] International Federation of Medical Students Association National Member Organisation Medsin UK. Access to Medical and Humanitarian Aid in Syria Policy Statement. International Federation of Medical Students Association Conference March meeting. Hammamet, Tunisia. 2014

[17] United Nations Security Council. Resolution 2139. Available at: http://www.un.org/en/ga/search/view_doc.asp?symbol=S/RES/2139%282014%29 Accessed: 22 March 2014.

